# Burden and outcomes of bacterial meningitis among children in tertiary hospitals in Niamey, Niger

**DOI:** 10.1016/j.ijregi.2026.100904

**Published:** 2026-04-21

**Authors:** Moussa Saley Sahada, Samaila Aboubacar, Souleymane Adoum Fils, Boulama Mamadou Boulama Malam, Garba Moumouni, Souley Nagoundi Ramatou, Adamou Bara Abdoul-Aziz, Kamaye Moumouni, Doutchi Mahamadou, Adehossi Eric, Serge Paul Eholié

**Affiliations:** 1 Faculty of Health Sciences of Abdou Moumouni University, Niamey, Niger; 2 Infectious Diseases Department, Niamey National Hospital, Niger; 3 Faculty of Health Sciences of André Salifou University, Zinder, Niger; 4 Infectious and Tropical Diseases Department at Zinder National Hospital, Niger; 5 Felix Houphouët-Boigny University, Abidjan, Ivory Coast

**Keywords:** Bacterial meningitis, Epidemiology, Child, Niger, Prognosis

## Abstract

**Objectives:**

Bacterial meningitis remains a major public health problem in Niger. Despite vaccination campaigns, the disease persists and continues to cause high morbidity and mortality in the pediatric population. This study aimed to describe the epidemiologic and evolutionary characteristics of bacterial meningitis in children in two tertiary hospitals in Niamey.

**Methods:**

This was a retrospective, descriptive, and analytical cross-sectional study conducted over a 22-month period (2023-2024). The study population included all children aged 0-59 months hospitalized for suspected or confirmed bacterial meningitis. Data were collected exhaustively from patient records. Variables associated with mortality in bivariate analysis were included in a multivariable logistic regression model. The Pearson chi-squared test at a significance level of 5% was used to determine factors associated with death by multivariate analysis.

**Results:**

Of a total of 220 suspected cases of meningitis, 105 (47.7%) were confirmed. The mean age was 24.2 ± 16.9 months, with a male-to-female ratio of 1.5. Fever (98.2%) and seizures (74.1%) were the main reasons for consultation. *Neisseria meningitidis* was the predominant pathogen (80.9% of cultures), with a majority of serogroup W135 identified by polymerase chain reaction (60%). The overall case fatality rate was alarming, reaching 50.9%, and 6.4% of survivors had sequelae. The main risk factors for death were age ≤24 months (odds ratio [OR] = 2.81), short duration of corticosteroid therapy (OR = 11.48), and antibiotic monotherapy.

**Conclusion:**

Pediatric bacterial meningitis in Niamey is characterized by high mortality and the predominance of *N. meningitidis.* These results call for an urgent review of national treatment protocols, the strengthening of diagnostic capacities, and the accelerated introduction of multivalent conjugate vaccines to cover emerging serogroups.

## Highlights

• Impact on infant mortality: bacterial meningitis remains a major cause of death in children under 5 years old in Niger, despite advances in vaccination.
• Epidemiological evolution: after the introduction of the vaccine against serogroup A, *Neisseria meningitidis* (including other serogroups) has become the dominant pathogen, illustrating a phenomenon of strain replacement.
• Critical seasonality: the disease burden peaks during the dry season, confirming the persistent vulnerability of populations in the African “meningitis belt.”
• Prognostic factors: delayed hospitalization is identified as the critical determinant of worsening clinical problems and increased case fatality rates.

## Introduction

Bacterial meningitis remains one of the most severe infectious diseases in pediatrics, constituting a medical emergency and a persistent global public health challenge. It is a major cause of childhood mortality and long-term neurologic disability worldwide, with the highest burden observed in low- and middle-income countries. Due to its epidemic potential, high lethality, and risk of devastating sequelae, bacterial meningitis continues to disproportionately affect young children, whose immune systems are still developing [1,2].

Despite substantial progress in prevention and treatment, including the widespread introduction of vaccines against *Haemophilus influenzae* type b and *Streptococcus pneumoniae*, bacterial meningitis remains responsible for significant morbidity, mortality, and health care costs, particularly, in sub-Saharan Africa. Worldwide, an estimated 1 million new cases of community-acquired bacterial meningitis occur each year, corresponding to an incidence of approximately 20 cases per 100,000 population. However, this burden is unevenly distributed, with low- and middle-income countries bearing the greatest impact. Investigating disease outcomes remains a research priority to identify modifiable risk factors and improve survival [3,4].

The burden of bacterial meningitis is particularly pronounced in the African “meningitis belt,” a vast region extending from Senegal to Ethiopia. This area is characterized by recurrent, often seasonal, epidemics that coincide with the dry season, during which dust-laden winds damage the respiratory mucosa and facilitate person-to-person transmission via respiratory droplets [5,6]. In this epidemiologic context, bacterial meningitis reaches exceptional severity, with case fatality rates that may exceed 70%. Even among survivors, nearly one in five children develops disabling long-term sequelae, including hearing loss, cognitive impairment, epilepsy, or motor paralysis [[7], [8], [9]].

Niger, located at the center of the meningitis belt, has historically experienced large-scale meningococcal epidemics. The introduction of the serogroup A conjugate vaccine (MenAfriVac) in 2010 marked a major turning point, leading to the near disappearance of *Neisseria meningitidis* serogroup A disease. However, this public health success has been followed by a phenomenon of serogroup replacement, with the emergence of non-A serogroups, particularly, serogroups C and W. Several studies conducted during the post–MenAfriVac transition period have reported that outbreaks are now predominantly and sometimes described as exclusively due to the *N. meningitidis* serogroup C [10,11]. Nevertheless, microbiological surveillance data remain limited, and uncertainties persist regarding the current epidemiologic profile of circulating strains. Microbiological confirmation of bacterial meningitis relies primarily on direct examination and culture of cerebrospinal fluid (CSF), whereas polymerase chain reaction (PCR) has significantly improved pathogen detection through its high sensitivity and specificity [[12], [13], [14], [15]]. Studies conducted between 2008 and 2015 in Niger documented a shift in meningococcal epidemiology after MenAfriVac introduction, with *N. meningitidis* serogroup A accounting for 54.2% of cases during the early post-vaccine period, followed by the emergence of serogroups W (24.2%) and C (21.1%) as major causes of outbreaks. More recent data suggest that meningitis epidemics in Niger are now primarily driven by serogroup C, although sporadic cases and outbreaks continue to occur despite ongoing vaccination efforts [8,16]. In response to this evolving epidemiologic landscape and the need for updated, locally generated evidence, this study was undertaken. Its objective was to describe the epidemiologic, clinical, microbiological, diagnostic, and therapeutic characteristics of bacterial meningitis in children aged 0-59 months who were hospitalized in two national reference hospitals in Niamey, Niger and identify factors that are independently associated with mortality and neurologic sequelae. By providing contemporary data on circulating pathogens and prognostic determinants, this work aims to inform public health policies and improve pediatric meningitis management in Niger.

## Methods

### Framework, type, and period of study

We conducted a multicenter cross-sectional study with descriptive and analytical aims, combining retrospective and prospective data collection over a total period of 22 months, from January 1, 2023 to October 31, 2024. The study was carried out in the pediatrics and infectious diseases departments of two tertiary referral hospitals in Niamey, Niger: the National Hospital of Niamey and the National Hospital Amirou Boubacar Diallo.

Both hospitals serve as national reference centers for pediatric and infectious disease care and provide services to urban populations in Niamey and the surrounding rural areas referred for specialized management.

The study comprised two phases:

▪ A retrospective phase from January 1, 2023 to December 31, 2023;
▪ A prospective phase from January 1, 2024 to October 31, 2024.

### Study population

The study population consisted of all children aged 0-59 months who were hospitalized in the participating departments during the study period with suspected or confirmed bacterial meningitis.

### Inclusion criteria

Children were eligible for inclusion if they met the following criteria:

▪ Age between 0 and 59 months;
▪ Hospitalization for suspected or confirmed bacterial meningitis;
▪ Provision of written informed consent by parents or legal guardians for participation in the prospective phase of the study.

### Non-inclusion criteria

Children were not included in the study if:

▪ Medical records were incomplete or insufficient for analysis;
▪ Parents or legal guardians refused to provide informed consent for the prospective phase.

### Case definitions

Case definitions were based on World Health Organization criteria:

▪ Suspected case: acute onset of fever (>38.5°C rectal or >38.0°C axillary), with at least one meningeal sign or bulging fontanelle in infants.
▪ Confirmed case: suspected case with bacterial identification in CSF by culture or PCR.
▪ Probable case: CSF cytology compatible with bacterial meningitis in the absence of microbiological confirmation.

The primary analysis focused on confirmed cases. Probable cases were included in descriptive analyses only.

### Data collection, laboratory investigations, and statistical analysis

Data were extracted from medical records, laboratory databases, and health booklets. Collected variables included sociodemographic data, clinical presentation, laboratory results, treatment, and outcomes. CSF was analyzed by cytology, Gram stain, culture, and, when available, PCR for *N. meningitidis* serogroups. Data were analyzed using Epi Info 7.2.6 and R. Categorical variables were compared using chi-square or Fisher’s exact tests. Multivariable logistic regression identified factors associated with mortality and neurologic sequelae; the results were expressed as adjusted odds ratios (ORs) with 95% confidence intervals (CIs). Statistical significance was set at *P* < 0.05.

Due to the tertiary referral nature of the study hospitals, only hospitalized patients were included. Data on children managed exclusively at primary health care facilities were not available, which may have led to an underestimation of the disease burden. Most patients were initially managed at primary health care facilities before referral, and antibiotic therapy was frequently initiated before hospital admission. Latex agglutination tests were not available; serogroup identification relied solely on PCR when performed. Variables associated with mortality in bivariate analysis were entered into a multivariable logistic regression model. Multicollinearity was assessed before model fitting.

## Results

### Sociodemographic aspects

**Figure 1:**
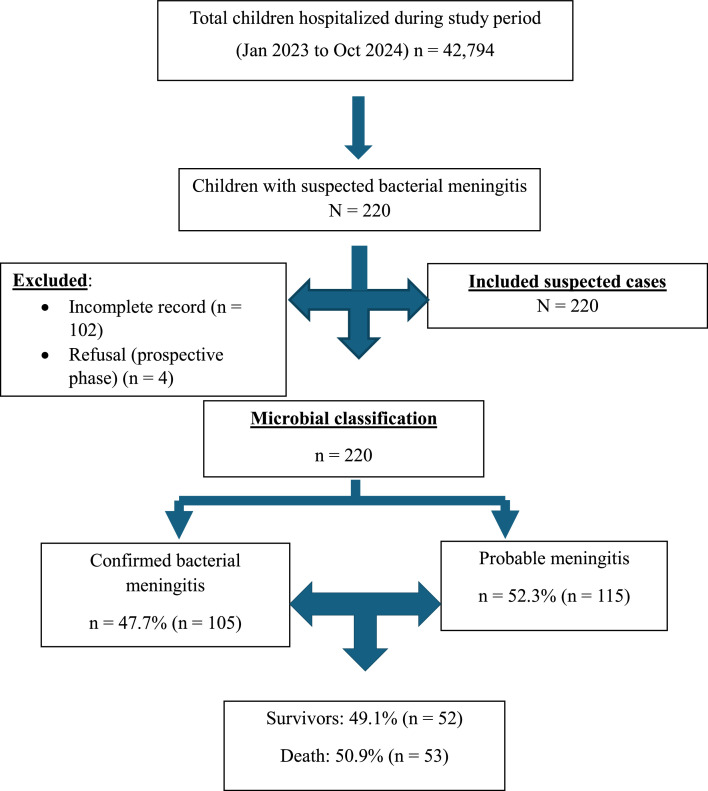
Flow diagram of patient inclusion.

During the 22-month study period, of the 42,794 children hospitalized in the two centers, 220 were included as suspected cases of bacterial meningitis. Among them, 105 cases were confirmed by cytological and/or bacteriological analysis of the CSF, representing 47.7%. The majority of patients, 72.2% of cases (n = 159), were registered at the Niamey National Hospital (Figure 1).

**Figure 2:**
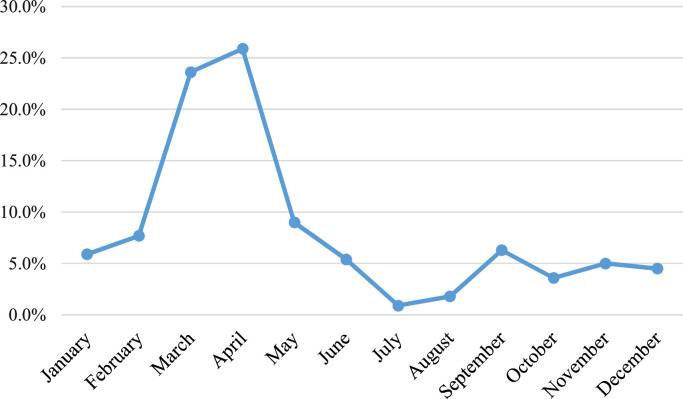
Distribution of patients according to month.

Over a 12-month period, analysis of the monthly distribution of cases revealed a marked seasonality, characteristic of the meningitis belt. The peaks in frequency were observed during the dry season, in March (23.6%) and April (25.9%); these two months accounting for nearly half of the annual cases (Figure 2).

Table 1 summarizes the main characteristics of the population of 220 children suspected of having bacterial meningitis. The mean age was 24.2 ± 16.9 months, with the majority of children aged 12-48 months (63.2%). The age groups <12 months and 12-48 months were the most represented, accounting for 29.1% and 63.1% of cases, respectively. There was a male predominance, with a sex ratio of 1.5. Most patients came from urban areas (74.5%). The average time before consultation was 4.3 days, and the main reasons for consultation were fever and seizures (Table 1).

**Table 1:** Epidemiologic, clinical, and microbiological characteristics data.

Epidemiology and clinical data (n = 220)	%	Paraclinical data	%
**Confirmed cases**	**47.7**	**Microbiology (confirmed cases, n = 105)**	
**Demographic characteristics**		*Neisseria meningitidis*	80.9
**Male sex**	60	*Haemophilus influenza*	10.5
**Urban residence**	74.5	*Streptococcus pneumoniae*	7.6
**Age range (months)**		*Listeria monocytogenes*	0.9
0-11	29.1		
12-48	63.2		
48-59	7.7	**PCR results (n = 25)**	
**Clinical**		*Neisseria meningitidis W135*	60
Fever	72.7	*Neisseria meningitidis X/W135*	4
Headaches	11.8	*Neisseria meningitidis X*	8
Vomiting	30.1	*Neisseria meningitidis C*	8
Convulsions	69.2	*Haemophilus influenza type b*	8
Altered consciousness	61.4	*Neisseria meningitidis Y/W135*	12

PCR testing was available for a subset of patients only.

Abbreviation: PCR, polymerase chain reaction.

A cloudy appearance was observed in 42.7% of patients. Gram-negative diplococci were the most frequent isolate on Gram staining, present in 81.9% of cases. *N. meningitidis* was the most frequently isolated organism in culture, accounting for 80.9% of cases. Serogroup W135 of *N. meningitidis* was the most frequently identified serogroup, accounting for 60% of PCR-confirmed cases. Monotherapy with a β-lactam antibiotic, such as ceftriaxone, was the standard treatment for over half of the patients (59.54%). Corticosteroid therapy was administered in 90% of cases. The immediate outcome was favorable in 49.1% of patients. Over half of the patients (70.91%) had a hospital stay of between 1 and 7 days. The average length of hospital stay was 5.1 ± 2.1 days, with a range of 1 to 24 days.

Bacteriological analysis of the 105 confirmed cases revealed a clear predominance of *N. meningitidis*. PCR testing was performed on 25 (11.3%) cases. More than half of the PCR results were positive for *N. meningitidis* W135 or 60% (n = 15) (Table 1).

Suspected cases without microbiological confirmation were classified as probable meningitis and managed empirically.

### Therapeutic and evolutionary aspects

All patients received ceftriaxone-based antibiotic therapy; 59.5% were treated with antibiotic monotherapy. Adjunctive corticosteroid therapy was administered in 90% of cases. The overall mortality among confirmed cases was 50.9%. Among survivors, 6.4% developed neurologic sequelae, including hemiplegia, epilepsy, visual impairment, and hydrocephalus (Table 2).

**Table 2:** Therapeutics and outcomes.

Treatment (n = 220)	%	Outcomes (n = 220)	%
Ceftriaxone	100	**Outcome**	
Dexamethasone	90	Favorable outcome (no sequelae)	42.7
Antipyretic	52.7	Sequelae	6.4
Antimalarials	45	Death	50.9
Diazepam	34.1	**Complication (18)**	
Oxygen therapy	17.7	Hemiplegia	27.8
Rehydration	8.2	Visual impairment	27.8
Phenobarbital	5.4	Epilepsy	16.7
		Encephalopathy	16.7
		Blindness	5.5
		Hydrocephalus	5.5

### Standard balance sheets

Computed tomography scans showed cerebral atrophy in two (66.7%) patients. Electroencephalogram revealed generalized epilepsy in 33.3% of cases (n = 1). Minimal triventricular hydrocephalus was found in 33.3% of cases (n = 1) on transthoracic echocardiography. The immediate outcome was favorable in 41.3% of patients (n = 91). It was marked by the occurrence of sequelae in 6.3% of cases; the overall mortality rate was 50.9%. Hemiplegia and decreased visual acuity were the most frequently observed complications, each occurring in 35.71% of cases.

### Multivariate analysis

In the multivariable analysis, the factors independently associated with death included age ≤24 months (adjusted OR [aOR] 2.81; 95% CI 1.60-4.92), short duration of corticosteroid therapy (1-2 days) (aOR 11.48; 95% CI 1.45-90.75), and antibiotic monotherapy (aOR 3.82; 95% CI 2.12-6.89) (Table 3).

**Table 3:** Distribution according to factors associated with the occurrence of death.

Associated factors	aOR (95% CI)	*P*-value
Urban residence	0.43 (0.42-2.08)	0.01
Length of stay (≤5 days)	0.46 (0.24-0.87)	0.02
Short corticosteroid duration (1-2 days)	11.48 (1.45-90.75)	0.00
Antibiotic monotherapy	3.82 (2.12-6.89)	0.00

Abbreviations: aOR, adjusted hazard ratio; CI, confidence interval.

### Factors associated with neurologic sequelae

Neurologic sequelae were more frequent in children older than 24 months and in those presenting with recurrent convulsions. Age >24 months and ≥3 convulsions per day remained independently associated with sequelae in the adjusted analysis (Table 4).

**Table 4:** Distribution according to factors associated with the occurrence of sequelae.

Associated factors	aOR (95% CI)	*P-*value
Number of seizures (≥3)	0.24 (0.06-0.83)	0.02
Length of stay (≤5 days)	0.53 (0.17-1.66)	0.27

Abbreviations: aOR, adjusted hazard ratio; CI, confidence interval.

## Discussion

The hospital prevalence of bacterial meningitis during the study period was 0.2%, a value lower than that reported in some sub-Saharan and North African countries, such as Gabon (0.3%) and Tunisia (9.2%) [17,18]. This low prevalence could be explained by the early treatment of Ear, Nose, and Throat infections and the impact of vaccination campaigns. However, the fact that the Niamey region is located in the Lapeyssonnie belt, characterized by recurrent meningitis epidemics, underscores that this disease remains a major public health problem. Socioeconomic conditions and cultural or religious practices can also delay access to care and promote the transmission of the disease [19]. The delay in hospital consultation (mean 4.3 days) likely reflects initial care seeking at primary health care facilities, where empirical antibiotic treatment is often initiated before referral.

A male predominance was observed (60% of cases; male-to-female ratio = 1.5), which could reflect biological or behavioral differences, although sex does not appear to be a determining factor in the development of meningitis [20,21]. Sex does not appear to be a factor influencing the occurrence of meningitis.

Clinically, fever was the most consistent symptom, reflecting the systemic response to the infection. Gastrointestinal symptoms, particularly, constipation (84.7%) and vomiting (100%), can be explained by the impact of inflammation on the digestive system and by smooth muscle hypertonia related to the neuroinflammatory response. The predominant neurologic signs, such as headaches, meningeal rigidity, photophobia, and sonophobia, reflect meningeal inflammation and the activation of pain signaling pathways by inflammatory mediators [22]

The CSF presented a variable macroscopic appearance, predominantly cloudy or purulent, reflecting a high bacterial load and an intense inflammatory response. The observation of clear CSF in some cases could result from the early performance of the lumbar puncture and the variability in treatment timing. Bacterial confirmation was obtained in 47.73% of cases, highlighting the limitations of conventional methods and the need for greater use of PCR to accurately identify pathogens [13,15,20].

Conventional diagnostic methods such as Gram stain and culture have limited sensitivity, particularly, in patients who received antibiotics prior to lumbar puncture. PCR provides improved diagnostic accuracy in such contexts.

The systematic administration of corticosteroids to all patients reflects the delays in microbiological confirmation and the desire to limit the excessive inflammatory reaction. Recommendations regarding corticosteroids are primarily applicable to pneumococcal meningitis, and their efficacy depends on early administration [21]. In this context, the association between short-term corticosteroid use and high mortality suggests that early discontinuation or late initiation compromises their protective effect, particularly, in severely ill patients. The association between short-duration corticosteroid therapy and increased mortality likely reflects late initiation or early discontinuation in severe cases rather than a direct causal relationship.

The observed mortality rate remains high, exceeding the 10% generally reported in Africa and Asia [23,24]. Several factors may explain this: delayed access to care, transportation difficulties, initial consultation with traditional healers, comorbidity with severe malaria, and suboptimal treatment strategies, including antibiotic monotherapy factors associated with increased mortality included age ≤24.2 months, rural origin, prolonged hospital stay, leukocytosis, and the use of monotherapy, reflecting the physiological vulnerability of infants and the constraints of the local health care system. The association between antibiotic monotherapy and mortality may reflect delayed initiation, previous antibiotic exposure, and disease severity at admission rather than treatment inadequacy alone.

Regarding sequelae, 6.4% of patients presented with neurologic complications at discharge, a figure likely underestimated due to the lack of systematic post-hospitalization follow-up. The pathophysiology of sequelae is based on a strong local inflammatory response induced by meningococcal endotoxins, with leukocyte infiltration, disruption of the blood–brain barrier, cell apoptosis, intravascular coagulation, and ischemia [23,24]. Age and the number of seizures were the main predictive factors for sequelae, whereas sex, vaccination status, or serogroup had no influence. Previous studies show that some sequelae can appear late, up to 60 months after infection. The observed male predominance may be related to differences in health-seeking behavior or exposure rather than biological susceptibility.

This study has several limitations. First, its hospital-based design may underestimate the true burden of disease at the community level. Second, previous antibiotic use before admission may have reduced microbiological confirmation rates. Third, PCR testing was not systematically available, limiting pathogen identification. Finally, the absence of population-based data prevented estimation of incidence rates.

This study has significant limitations, including the limited use of PCR, the lack of systematic follow-up of long-term sequelae, and the possible underestimation of severe cases that did not reach the hospital. Nevertheless, these results highlight the persistent severity of pediatric bacterial meningitis in Niger and the need to strengthen early intervention, optimize treatment protocols, and organize structured post-hospitalization follow-up for surviving children.

## Conclusion

Meningitis among infants and children poses a serious public health problem because it is a major cause of morbidity and mortality. However, the outcome was fatal in more than half of the cases. In light of these findings, this disease warrants particular attention and a focus on prevention and appropriate treatment. The results of this study provide sufficient evidence of the emergence of meningococcal C cases. The fight against these types of meningitis requires improvements in epidemiologic surveillance to detect small changes in the distribution of serogroups. This surveillance could have significant implications for public health strategies in the coming seasons, especially with the introduction of the Men5Cv conjugate vaccine in Niger.

## Declaration of competing interest

The authors have no competing interests to declare.
